# Policy impact of the Imperial College COVID-19 Response Team: global perspective and United Kingdom case study

**DOI:** 10.1186/s12961-024-01236-1

**Published:** 2024-11-13

**Authors:** Sabine L. van Elsland, Ryan M. O’Hare, Ruth McCabe, Daniel J. Laydon, Neil M. Ferguson, Anne Cori, Paula Christen

**Affiliations:** 1 MRC Centre for Global Infectious Disease Analysis, School of Public Health, Imperial College London, London, United Kingdom; 2 Center for Epidemiological Modelling and Analysis (CEMA), University of Nairobi, Nairobi, Kenya; 3 Communications Division, Imperial College London, London, United Kingdom; 4 Jameel Institute, School of Public Health, Imperial College London, London, United Kingdom

**Keywords:** Knowledge translation, Evidence-to-policy pathway, COVID-19, Mathematical modelling

## Abstract

**Background:**

Mathematical models and advanced analytics play an important role in policy decision making and mobilizing action. The Imperial College Coronavirus Disease 2019 (COVID-19) Response Team (ICCRT) provided continuous, timely and robust epidemiological analyses to inform the policy responses of governments and public health agencies around the world. This study aims to quantify the policy impact of ICCRT outputs, and understand which evidence was considered policy-relevant during the COVID-19 pandemic.

**Methods:**

We collated all outputs published by the ICCRT between 01-01-2020 and 24-02-2022 and conducted inductive thematic analysis. A systematic search of the Overton database identified policy document references, as an indicator of policy impact.

**Results:**

We identified 620 outputs including preprints (16%), reports (29%), journal articles (37%) and news items (18%). More than half (56%) of all reports and preprints were subsequently peer-reviewed and published as a journal article after 202 days on average. Reports and preprints were crucial during the COVID-19 pandemic to the timely distribution of important research findings. One-fifth of ICCRT outputs (21%) were available to or considered by United Kingdom government meetings. Policy documents from 41 countries in 26 different languages referenced 43% of ICCRT outputs, with a mean time between publication and reference in the policy document of 256 days. We analysed a total of 1746 policy document references. Two-thirds (61%) of journal articles, 39% of preprints, 31% of reports and 16% of news items were referenced in one or more policy documents (these 217 outputs had a mean of 8 policy document references per output). The most frequent themes of the evidence produced by the ICCRT reflected the evidence-need for policy decision making, and evolved accordingly from the pre-vaccination phase [severity, healthcare demand and capacity, and non-pharmaceutical interventions (NPIs)] to the vaccination phase of the epidemic (variants and genomics).

**Conclusion:**

The work produced by the ICCRT affected global and domestic policy during the COVID-19 pandemic. The focus of evidence produced by the ICCRT corresponded with changing policy needs over time. The policy impact from ICCRT news items highlights the effectiveness of this unique communication strategy in addition to traditional research outputs, ensuring research informs policy decisions more effectively.

## Background

Scientific evidence from mathematical models and advanced analytics play an important role in policy decision making [1, 2] and mobilizing action during disease outbreaks [3]. For example, epidemiological models can provide insight into the number of cases at a given time, disease severity (e.g. expected hospitalizations and deaths), the rate that a disease spreads through a population, and the final size of an epidemic. Models can further be used to explore scenarios, such as the likely impact of interventions [4]. Such models are particularly important for novel viral outbreaks, where there is considerable uncertainty [5].

Evidence-informed decision making is also referred to as knowledge translation. This can be defined as the synthesis, exchange and application of knowledge by relevant stakeholders to accelerate the benefits of global and local innovation in strengthening health systems and improving people’s health [6]. The coronavirus disease 2019 (COVID-19) pandemic motivated the generation of scientific evidence at remarkable speed and scale, receiving unprecedented national and international attention outside of academia [7]. Some countries had more established modelling capacity and pre-established data-to-decision pathways than others [8]. The variability in countries’ pandemic responses resulted in an ongoing natural experiment of how scientific evidence, public health and policy decisions, as well as political actions influence the trajectory of the global crisis [9].

In the United Kingdom, the Scientific Pandemic Influenza group on modelling operational subgroup (SPI-M-O) collated results and insights generated by multiple independent modelling groups and experts to provide a consensus position. This scientific evidence was made available to the United Kingdom’s Government’s Scientific Advisory Group for Emergencies (SAGE), which in turn informed policy [10].

The Imperial College COVID-19 Response Team (ICCRT) at the Medical Research Council (MRC) Centre for Global Infectious Disease Analysis, Imperial College London (textbox 1) and other research groups produced a large body of evidence to inform policy decision making. To improve preparation and protection against new emerging threats, it is important to understand how these efforts facilitate the evidence-to-policy pathway. This study aims firstly to quantify the policy impact of the work by the ICCRT, and secondly to understand the themes of evidence developed by the ICCRT during the COVID-19 pandemic that were most relevant for policy makers – in other words, what defined policy-relevant evidence.

[Textbox 1]: Context The MRC Centre for Global Infectious Disease Analysis is a World Health Organization collaborating centre for Infectious Disease Modelling. Established in 2007, it builds on well-established global partnerships and extensive experience in previous infectious disease outbreaks including the bovine spongiform encephalopathy (BSE) [11] and Creutzfeldt-Jakob disease (vCJD) [12] epidemic in the 1990s, the foot-and-mouth epidemic [13], avian influenza [14] and pandemic influenza [15] in the early 2000s. Since its founding, the MRC Centre has undertaken real-time research on the 2009 H1N1 influenza pandemic [47], Middle East respiratory syndrome coronavirus (MERS-CoV, 2013-) [18], Ebola (2014-) [16], Zika (2016) [17], severe acute respiratory syndrome coronavirus 2 (SARS-CoV-2, 2020-) [29] and MPOX virus (2022-) [48]. The MRC Centre’s Imperial College COVID-19 Response Team (ICCRT) was one of the expert groups providing evidence to the United Kingdom’s Scientific Pandemic Influenza group on modelling operational subgroup (SPI-M–O). In addition, the ICCRT also provided epidemiological analysis to inform the policy response of governments and public health agencies globally [19]

## Methods

We collated all outputs published by members of the ICCRT [including Real-time Assessment of Community Transmission (REACT) study outputs [20]] between 01-01-2020 and 24-02-2022, when all domestic legal COVID-19 restrictions in England were lifted [21]. Reports, preprints and journal articles were identified through Imperial’s institutional open access research repository, Spiral. Reports were categorized as: self-published (published through Spiral, authored by the ICCRT), commissioned (publicly released externally by United Kingdom government, authored by the ICCRT), contributed to (publicly released externally by United Kingdom government or Academy of Medical Sciences, co-authored by at least one member of the ICCRT), or a consensus statement [10] (publicly released externally by United Kingdom government, reporting the combined consensus estimate using a range of models including those from the ICCRT). We identified outputs produced or co-authored by the ICCRT that were publicly available through gov.uk, i.e. the collection of scientific evidence supporting the United Kingdom government response to COVID-19 [23]. This collection also identified all other outputs by the ICCRT (self-published, preprint servers or peer-reviewed scientific journals) which were made available or considered as evidence at SAGE meetings, by the United Kingdom Government Chief Scientific Adviser (GCSA) and their deputies, or Chief Medical Officer (CMO).

Imperial news items on ICCRT outputs (including reports, preprints, journal publications and software) were identified through the Imperial news pages [24]. We excluded any miscellaneous news items including those on events, awards, question and answers (Q and As) or perspective pieces.

Inductive thematic analysis of all outputs was conducted by two authors (S.L.v.E., R.O.H.); discrepancies were resolved by consensus. Related outputs (e.g. preprint or report and a subsequent peer-reviewed publication in a scientific journal, or the corresponding news item dedicated to the specific output) were cross-referenced to ensure the same theme was applied to each output type.

The number of policy documents that cite research outputs is a commonly used metric used as an indicator of policy impact [25, 26]. This measure was collected via the Overton database, which includes over 12 million policy documents from governments, official bodies, intergovernmental organizations (IGOs) [e.g. the World Health Organization (WHO)] and think tanks from nearly 200 countries [25]. All outputs by the ICCRT which were made public by the United Kingdom government were excluded from this part of the analysis, as these can be considered policy transfer [27] (policy-to-policy translation or the adoption and/or adaptation of policy foreign to the decision-making context) instead of policy impact (evidence-to-policy translation). The Overton search was performed using the DOI for reports, preprints and journal articles. For news items, the search was performed using the uniform resource locator (URL) both with and without the scheme, subdomain or domain (‘https://’, ‘www.’ and ‘imperial.ac.uk/news/’). All Overton reports were extracted on 22-01-2024.

We distinguished between the first phase (pre-vaccination 01-01-2020 until 30-11-2020) and the second phase (vaccination available 01-12-2020 till 28-02-2022) of the pandemic. This division is based on the vaccination roll-out of the United Kingdom government [28]. Pearson chi-squared test (*χ*^2^) and independent samples *t*-test with *P* values (significance measured at *p* < 0.05) were used to compare outputs and policy impact across pandemic phases and themes.

## Results

We identified 620 outputs including 97 preprints (16%), 180 reports (29%), 231 journal articles (37%) and 112 news items (18%) between 1 January 2020 and 28 February 2022.

Most preprints (94%) were published on medRxiv. Two-thirds of the reports were publicly released by the United Kingdom government (*n* = 116, 64%), of which 35 (30%) were commissioned reports, 19 (16%) were consensus statements and 62 (54%) were reports the ICCRT contributed to. A small number of reports were duplicated on preprint servers (7 of 180, 4%).

Ten reports were self-published by the REACT study, and of the other 51 self-published ICCRT reports, 33 (65%) were subsequently peer-reviewed and published as a journal article. More than half (56%) of all reports and preprints combined (excluding United Kingdom government-released reports) were subsequently peer-reviewed and published as a journal article (90 of 161). The mean time from report or preprint to journal publication was 202 days [5–746 days, median 187 days, interquartile range (IQR) 82.8–285.0] (Table 1).

**Table 1:** ICCRT output format and policy references

	Total *N* = 620	Preprints *N* = 97	Reports *N* = 180	Journal articles *N* = 231	News items *N* = 112
	*n* (%)	*n* (%)	*n* (%)	*n* (%)	*n* (%)
Subsequently peer-reviewed	90 (67.5)	57 (58.8)	33 (18.3)	–	–
ICCRT reports only	–	–	33 (64.7)**	–	–
Time (days) to publication, mean (min–max)	201.8 (5–746)*	187.9 (5–746)	225.7 (35–596)	–	–
Available or considered at United Kingdom government meeting	130 (21.0)	2 (2.1)	128 (71.1)	0 (0)	0 (0)
Referenced in policy document(s)	217 (43.1)^	38 (39.2)	20 (31.3)^^	141 (61.0)	18 (16.1)
Time (days) to policy publication, mean (min–max)	255.7 (−48–1348.0)^	211.8 (2–948)	172.5 (1–1319)^^	272.0 (−48–1348)	210.2 (35.0–1001.0)
Number of policy references per output, mean (min–max)	8.1 (1–119)^	4.1 (1–25)	8.5 (1–69)^^	9.8 (1–119)	2.0 (1–11)
Time-period					
First phase (pre-vaccination, Jan-Nov 2020)	273 (44.0)	45 (16.5)	89 (32.6)	75 (27.5)	64 (23.4)
Second phase (vaccination, December 2020–February 2022)	347 (56.0)	52 (15.0)	91 (26.2)	156 (45.0)	48 (13.8)

^*^N = 277 only including reports and preprints, **N = 51 only including ICCRT self-published reports (excluding REACT published reports and United Kingdom government-published reports), ^*N* = 504 only including non-government published outputs, ^^*N* = 64 only including non-government published outputs

To identify which outputs constituted policy relevant evidence, we identified 13 themes across the 620 outputs. The most frequent themes were: (1) severity, healthcare (HC) demand and capacity (*n* = 123, 20%); (2) non-pharmaceutical interventions (NPIs) (*n* = 96, 16%); (3) surveillance and testing (*n* = 90, 15%); and (4) variants and genomics (*n* = 82, 13%) (Table 2).

**Table 2:** ICCRT outputs and policy references by theme and phase of pandemic

	Outputs *N* = 620	Phase 1 *N* = 273	Phase 2 *N* = 347	Pearson *χ*^2^ (*P* value)	Policy reference *N* = 1746	Phase 1 *N* = 1099	Phase 2 *N* = 647	Pearson *χ*^2^ (*P* value)
	*n* (%)	*n* (%)	*n* (%)		*n* (%)	*n* (%)	*n* (%)	
Severity/HC demand and capacity	123 (19.8)	54 (19.8)	69 (19.9)	0.001 (0.974)	269 (15.4)	218 (19.8)	51 (7.9)	44.650 (< 0.001)*
Non-pharmaceutical interventions (NPIs)	96 (15.5)	42 (15.4)	54 (15.6)	0.004 (0.952)	453 (25.9)	342 (31.1)	111 (17.2)	41.325 (< 0.001)*
Surveillance/testing	90 (14.5)	38 (13.9)	52 (15.0)	0.140 (0.708)	147 (8.4)	84 (7.6)	63 (9.7)	2.316 (0.128)
Variants/genomics	82 (13.2)	21 (7.7)	61 (17.6)	13.014 (< 0.001)*	264 (15.1)	63 (5.7)	201 (31.1)	203.658 (< 0.001)*
Transmission	48 (7.7)	27 (9.9)	21 (6.1)	3.151 (0.076)	102 (5.8)	52 (4.7)	50 (7.7)	6.647 (0.010)*
Secondary impact	44 (7.1)	24 (8.8)	20 (5.8)	2.124 (0.145)	187 (10.7)	99 (9.0)	88 (13.6)	8.984 (0.003)*
Vaccines	32 (5.2)	5 (1.8)	27 (7.8)	11.049 (< 0.001)*	66 (3.8)	28 (2.5)	38 (5.9)	12.382 (< 0.001)*
Outbreak scale/under-ascertainment	28 (4.5)	24 (8.8)	4 (1.2)	20.674 (< 0.001)*	134 (7.7)	118 (10.7)	16 (2.5)	39.252 (< 0.001)*
Health systems/policy	19 (3.1)	6 (2.2)	13 (3.7)	1.233 (0.267)	14 (0.8)	5 (0.5)	9 (1.4)	4.486 (0.034)*
Other	18 (2.9)	11 (4.0)	7 (2.0)	2.194 (0.139)	7 (0.4)	5 (0.5)	2 (0.3)	0.217 (1.000)^
Behaviour/mobility	16 (2.6)	11 (4.0)	5 (1.4)	4.072 (0.044)*	80 (4.6)	71 (6.5)	9 (1.4)	23.938 (< 0.001)*
Clinical/treatment	13 (2.1)	6 (2.2)	7 (2.0)	0.024 (0.876)	12 (0.7)	11 (1.0)	1 (0.2)	4.274 (0.039)*
Economics	11 (1.8)	4 (1.5)	7 (2.0)	0.267 (0.605)	11 (0.6)	3 (0.3)	8 (1.2)	6.039 (0.014)*

^*^ Significant (*P* < 0.05), ^Fisher’s exact test considering outputs published in one theme compared to all other outputs and the difference between the two phases

### Evidence supporting the United Kingdom government

One-fifth of all ICCRT outputs (21%) were available to, or considered by, the United Kingdom GCSA, CMO or at SAGE meetings. These were reports (*n* = 128) and preprints (*n* = 2) only; no journal articles nor news items were considered in such a way (Table 1). The majority (*n* = 116) of these outputs were commissioned or contained contributions from the ICCRT authors to reports released by the United Kingdom government. The other outputs (*n* = 14) were published by the ICCRT as a self-published reports and preprints. The themes of evidence produced by the ICCRT for this subset of outputs which was considered by the United Kingdom government were (1) severity, HC demand and capacity (51%); (2) NPIs (29%); and (3) variants and genomics (8%).

For the purpose of further analysis in this paper, these outputs released by the United Kingdom government only (*n* = 116) are considered as an indicator of policy transfer [27] rather than policy impact. All 504 non-government-published outputs were included in further policy impact analysis.

### Global policy and ICCRT evidence

**Fig. 1:**
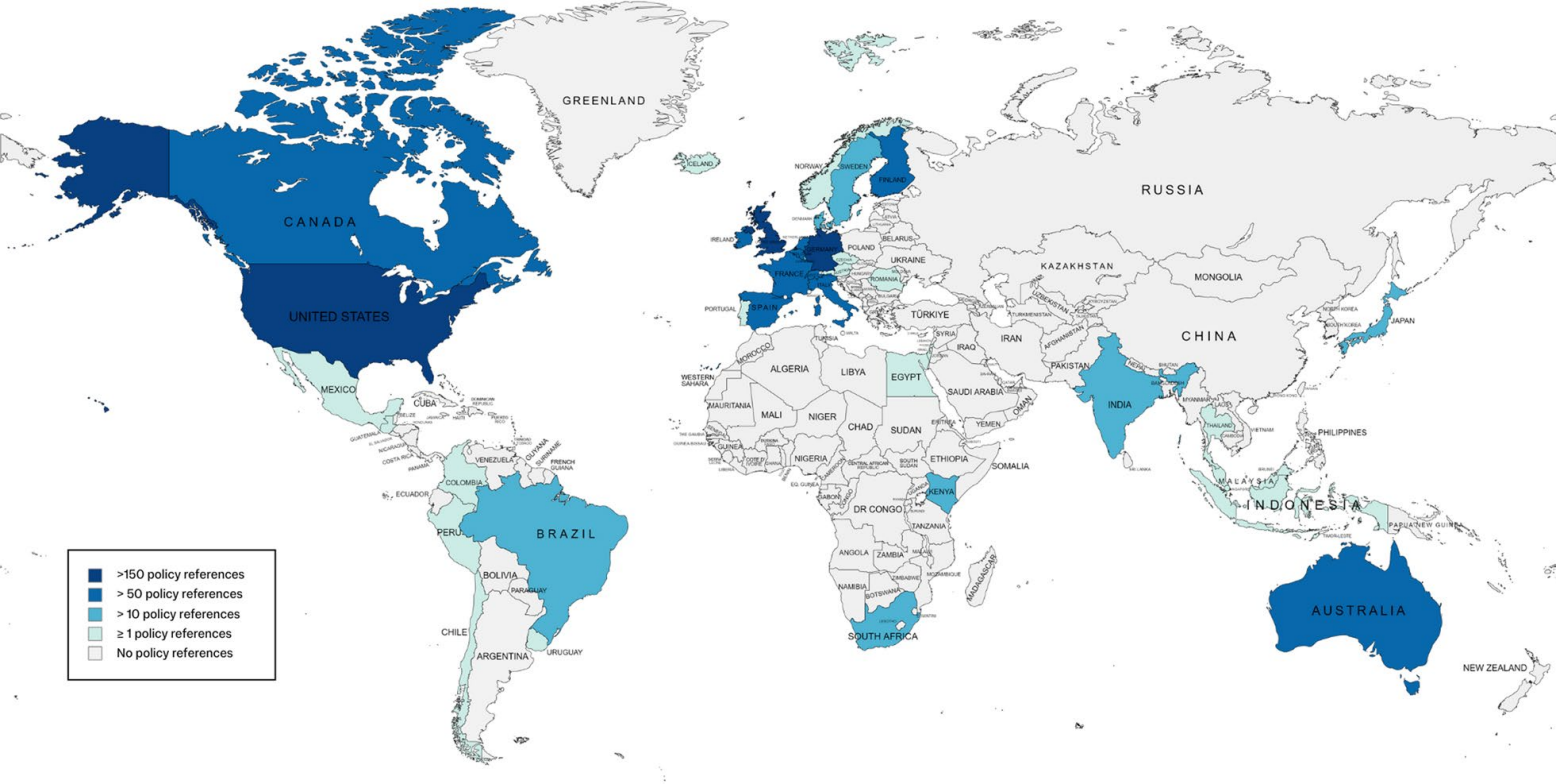
Policy impact by country. Overview of countries with one or more policy references, darker shade indicating more policy documents referencing ICCRT outputs. Europe (*n* = 87 policy references) and intergovernmental organizations (IGO) (*n* = 448 policy references) are not indicated on this map (map created with mapchart.net).

The number of references in policy documents globally served as the metric of policy impact in this study. We find that 43% of outputs were referenced in one or more policy documents from 41 countries or regions in 26 different languages (Fig. 1). These 217 outputs together had 1746 policy document references (1344 unique policy documents) and a mean of 8 policy documents per output, ranging between 1 and 119 documents. The output with 119 policy document references focussed on severity, healthcare (HC) demand and capacity, and was published very early in the pandemic [29]. These policy documents originated from 16 countries in 6 languages and were mostly think-tank documents (45%), government documents (25%) and documents from intergovernmental organizations (IGOs) (23%).

Similar to the United Kingdom government-released evidence, the themes of the evidence produced by the ICCRT corresponded with the policy need for evidence. The most frequent themes of outputs referenced in policy documents were (1) NPIs (26%); (2) severity, healthcare demand and capacity (15%); and (3) variants and genomics (15%) (Table 2).

### Output format to inform policy

In the context of the pandemic, we explore the effectiveness of several formats of communicating scientific evidence. All output formats were referenced in one or more policy documents, almost two-thirds (61%) of journal articles, 39% of preprints, one-third (31%) of non-government published reports and 16% of news items. Reports and journal articles were referenced in more policy documents (mean 8.5 and 9.8, respectively) than preprints and news items (mean 4.1 and 2.0, respectively) (Table 1).

### Evidence-to-policy delay

On average, there were 256 days (8 months) between output publication and referencing in policy documents. However, there were large variations. Less than one-quarter (23%) of outputs were referenced in policy documents within two months of publication, whereas the longest delay was 1348 days (44 months). Ten policy documents had a publication date on or before the date of the referenced output. In these cases, an online update of the policy document included a reference to an ICCRT output, or an ICCRT author was co-author on the policy document which were published in the United Kingdom, United States, Australia and by an IGO. The mean time between publishing and policy reference was shorter for reports (173 days, *P* < 0.003), news items (210 days, *P* = 0.075), and preprints (212 days, *P* < 0.001) and longer for journal articles (272 days, *p* < 0.001) compared with other outputs (Table 1).

### Changing evidence needs for policy

As the pandemic evolved, the policy needs for evidence changed. We considered the format of outputs produced and quantified the number of policy references from evidence in both phases of the pandemic (i.e. pre-vaccination and where vaccination was available).

**Fig. 2:**
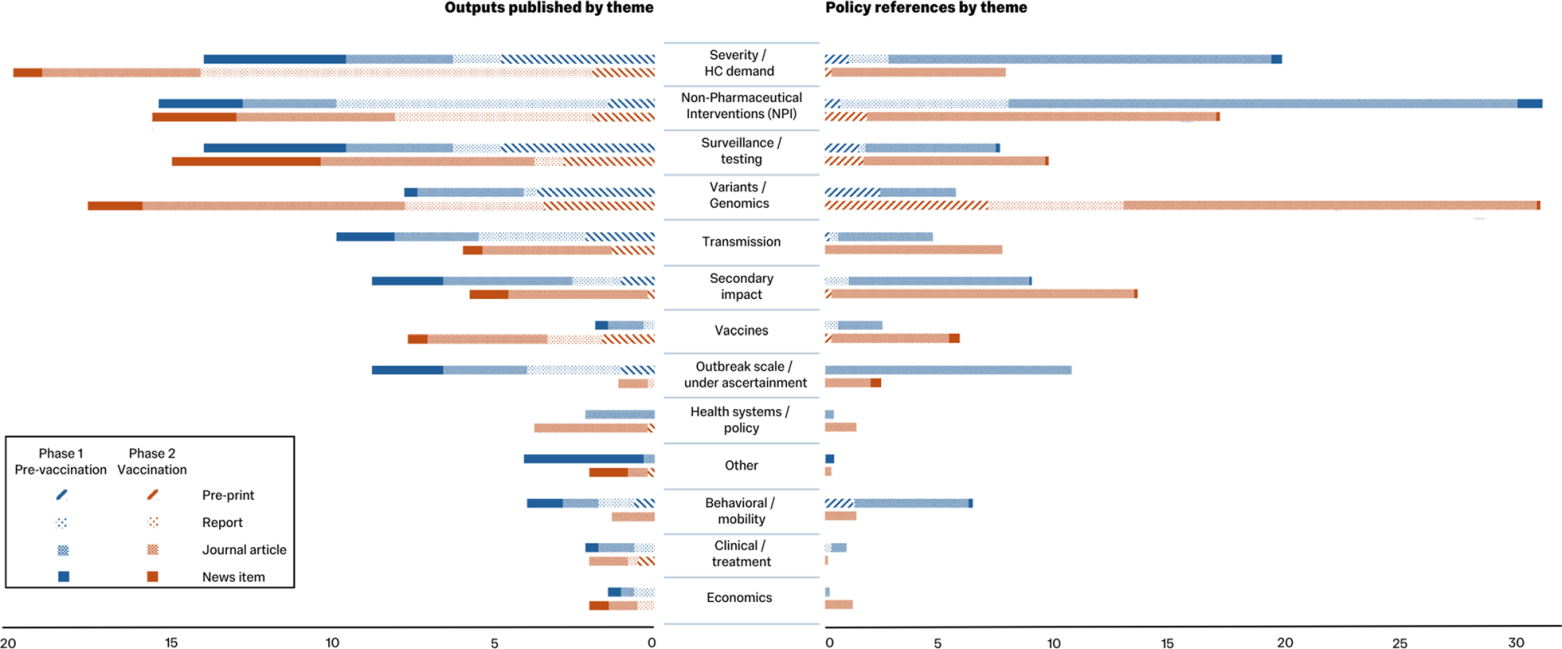
Outputs published and policy references by theme. Proportion (%) of outputs published (left) and policy references (right) by theme, stratified by format of output (preprint, report, journal article or news item

More news items were published in the first phase (23%) than the second phase (14%, *P* = 0.002). More journal articles were published in the second phase (45%) than the first phase (28%, *p* < 0.001). Preprints were more likely to have one or more policy references in the second phase (12%) than the first phase (8%, *p* = 0.004). We found that reports were more likely to be referenced in one or more policy document in the first phase (12%) than the second phase (6%, *p* < 0.001) (Table 1, Fig. 2).

### What constitutes policy-relevant evidence?

Next, we compared the themes of evidence produced by ICCRT in the first phase with the second phase. The most frequent themes of evidence produced were similar to the themes of the outputs that were referenced in policy documentation. The most produced and cited themes changed from the first to the second phase. During the first phase of the pandemic, ICCRT published most outputs on severity, HC demand and capacity (20%), and NPIs (15%). These exact same themes were the most referenced policy documents during this first phase with NPIs (31%), and severity, HC demand and capacity (20%). During the second phase ICCRT produced most outputs within the themes of severity, HC demand and capacity (20%), and variants and genomics (18%), with the most referenced themes in policy documentation being variants and genomics (31%),and NPIs (17%) (Table 2, Fig. 2).

During the first phase, ICCRT published more on outbreak scale and under-ascertainment (9%), and behaviour/mobility (4%), compared with the second phase (1%, *P* < 0.001 and 1%, *P* = 0.044, respectively). These themes were more likely to be referenced in policy documents in the first phase (11% and 7%, respectively) compared with the second phase (3%, *p* > 0.001 and 1%, *P* > 0.001, respectively) (Table 2).

During the second phase the ICCRT published more on variants and genomics (18%) and vaccines (7.8%) compared with the first phase (8% and 2%, respectively) (Table 2, Fig. 2). Similarly, references in policy documents to outputs on variants and genomics (31%),and vaccines (6%) occurred more often in the second phase compared with the first phase (6%, *P* > 0.001, and 3%, *P* > 0.001, respectively) (Table 2).

## Discussion

We explored the impact of outputs by the Imperial College COVID-19 Response Team on policy decision making, considering a wide range of research outputs and publication formats. We showed that the ICCRT’s evidence had wide-reaching impact on policy decision making globally, with 1746 policy document references across 41 countries. The most frequent themes of evidence produced reflected the evidence need for policy decision making, which evolved accordingly between the first phase (severity, HC demand and capacity, and NPIs) and the second phase of the pandemic (variants and genomics).

We found that two-thirds (64%) of the ICCRT reports were subsequently released by the United Kingdom government as supporting evidence in their decision-making process. The United Kingdom documented the breadth of evidence available to political decision-makers in government reports and had a dedicated SAGE which was a sub-committee of the Civil Contingencies Committee (COBRA) to support the emergency response [30–32]. In other countries, there was less transparency on which evidence informed political decisions during the pandemic. A recent workshop brought together global experts in advanced analytics and public health decision-makers to identify opportunities to strengthen the data-to-decisions pathways. They identified the need for a dynamic, transparent, equitable and accountable governance system. In many countries, evidence that informed policy was neither made available nor communicated transparently to the public, with policy decision-makers gatekeeping evidence. Scientists in some countries were not permitted to communicate the extent of uncertainties or explain knowledge gaps [33].

While mathematical models are widely used to inform public health decisions, their inherent uncertainties are often poorly communicated [34]. Policymakers often had poor understanding of key concepts, such as exponential growth and the limitations of long-term forecasting [35]. Two-way communication between modellers and policymakers is a critical factor in ensuring that suitable scenarios are modelled and results are understood [36, 37].

The results of this study show that the themes of outputs produced by the ICCRT and policy-relevant output themes shifted accordingly during the course of the pandemic. In the first phase of the pandemic ICCRT outputs and policy need for evidence focussed on severity, HC demand and capacity and NPIs. Focus then shifted to viral variants and genomics during the second phase after the introduction of vaccination. Such shift in key public health questions and corresponding data needs is common to many epidemics: moving from the need to understand how dangerous a pathogen is at the beginning phase of an epidemic, to monitor its impact, evolution and how to control the epidemic in the later phases [38]. Modelling approaches evolved on the basis of data availability and policy needs at different phases of the pandemic. When data were scarce early in the pandemic, most modelling centred on estimate key parameters of interest, such as the reproduction number and the infection fatality ratio. This focus shifted as more data became available, and transmission models were used to explore the impact of interventions and transitioning out of the emergency phase, modelling was used to examine effectiveness of policies made throughout the pandemic [36].

In a public health emergency, time-critical information with immediate public health implications must be rapidly disseminated without concern for subsequent consideration for publication in a journal [39]. Public reports and preprints were therefore crucial during the COVID-19 pandemic to the timely distribution of important research findings advancing our understanding of severe acute respiratory syndrome coronavirus 2 (SARS-CoV-2) [40]. Preprints democratize scientific publishing, as they are more equitably distributed across countries and income groups than publications [41]. We found 65% of the ICCRT self-published reports and 56% of all reports and preprints (majority on medRxiv) were peer-reviewed and published after a median of 187 days. A recent review by Eckmann et al*.* found 37% of all medRxiv preprint publications were subsequently peer-reviewed and published in a scientific journal, with a median of time gap between preprint and paper publication of 199 days [41]. Some journals accelerated their peer-review and publication schedules during the COVID-19 pandemic [42]; however, compared with the average preprint, ICCRT preprints and reports were more likely to subsequently be peer-reviewed and were published with a shorter delay. This is testament to the scientific rigour of the work and academic commitment of the ICCRT and contrasts with the frequent criticism of reduced academic rigour or quality in preprints [43, 44]. Given the most cited output format for policy documentation were journal articles, this likely benefited the evidence-to-policy pathway.

Although COVID-19 forecasting and modelling of public health responses has been heavily dependent on partnerships with academic research teams, university-based modellers face considerable barriers when choosing to engage in crucial, but time-consuming, translational work, e.g. building, maintaining and communicating modelling results. Extant incentive structures do not fully recognize these efforts, and instead reward traditional forms of academic achievement (e.g. peer-reviewed publications and secured grant funding). The value of this type of translational work needs to be recognized and elevated to continue the academic community’s engagement in real-time outbreak mitigation and maximize its impact [45].

Policy citations are shaped by a complex interplay between scientific research and its impact on society through policy discourse. Science communication channels such as news media and social media play a crucial role bridging the gap between research and policy [46]. Although more news items were published during the first phase, their policy impact did not waver in the second phase. The policy impact from ICCRT news items highlights the effectiveness of this unique communication strategy in addition to traditional research outputs, enabling research informing policy more effectively.

The English summaries of all 51 ICCRT self-published reports were translated into six additional languages (French, Spanish, Italian, Japanese, Mandarin and Arabic), with some summaries available in Portuguese and Bahasa Indonesian additionally. The ICCRT worked with academic partners, NGOs and health ministries in several countries spanning Latin America (e.g. Brazil, Columbia, Panama), Africa (e.g. Malawi, Nigeria, Senegal, Sudan, Zimbabwe) and Asia (e.g. India, Indonesia, The Philippines) to support country responses during the early stages of the pandemic [19]. It is likely that these efforts helped to make the ICCRT outputs more widely accessible resulting in references in policy documentation in some instances (e.g. Brazil and Indonesia). However, the results from this study suggest that some gaps remain in the global accessibility of the evidence produced by the ICCRT.

During the COVID-19 pandemic, many groups provided insight into the unfolding outbreak using mathematical modelling. These groups applied a variety of methodologies, had different work structures, resources and external networks which impacted policy differently. In this study, we present a case study using thematic analysis and policy citations as a metric for policy impact of the ICCRT research. The methodology presented in this paper can guide further research to understand this diversity and trends across academic groups providing scientific evidence for policy decision making.

Although research outputs referenced in policy documentation is a commonly used impact indicator [25, 26], using a single metric is a limitation of this study. Informal data-to-decision pathways and policy-to-policy translation (outputs publicly released by the United Kingdom government) were not explored in this study. The estimates presented in this study are therefore likely a conservative quantification of the impact of evidence on policy. However, ICCRT research could have been used to justify decisions that policy makers intended to make regardless of the evidence. It is a limitation of the current study that no distinction is made between instrumental, conceptual and symbolic evidence use. In addition, the impact of knowledge translation via (social) media outlets and journalists as evidence brokers were not evaluated beyond the Imperial news items analysed in this study. Similarly, this study did not reflect on the contribution of existing pathways, relationships and networks to the impact of evidence on policy decision making. Further research is required to better understand these pathways to improve transparent and bidirectional communication and prepare an effective response to future public health emergencies.

## Conclusion

This study focussed on the mathematical modelling outputs of the Imperial College COVID-19 Response Team (ICCRT) and its impact on domestic and global health policy. Policy-relevant evidence and changes in policy needs identified can help direct focus and resources more effectively during a future public health emergency of international concern.

Evidence produced by the ICCRT had a wide-reaching impact on policy decision making both in the United Kingdom and globally. The most frequent themes of outputs reflected the evidence needed by policymakers and evolved accordingly from the first phase to the second phase of the epidemic.

We find that the communication format was relevant to the impact of the work on policy. Public reports and preprints were crucial during the COVID-19 pandemic to the timely distribution of important research findings. Communication channels that were established during the pandemic can be leveraged for future response strategies. Further research is required to better understand informal data-to-policy pathways, improve transparent and bidirectional communication and prepare an effective response to future public health emergencies.

## Data Availability

The datasets generated and/or analysed during the current study are not publicly available but are available from the corresponding author on reasonable request.

## Abbreviations

COBR(A): Civil contingencies committee (Cabinet Office Briefing Rooms A)
COVID-19: Coronavirus disease
HC: Healthcare
ICCRT: Imperial College COVID-19 Response Team
IGO: Intergovernmental organization
MERS-CoV: Middle East respiratory syndrome coronavirus
MRC: Medical research council
NPIs: Non-pharmaceutical interventions
REACT: Real-time assessment of community transmission
SAGE: Scientific advisory group for emergencies
SARS-CoV-2: Severe acute respiratory syndrome coronavirus 2
SPI-M–O: Scientific pandemic influenza group on modelling operational subgroup
WHO: World Health Organization
